# Distribution Levels of Particulate Matter Fractions (<2.5 µm, 2.5–10 µm and >10 µm) at Seven Primary Schools in a European Ceramic Cluster

**DOI:** 10.3390/ijerph18094922

**Published:** 2021-05-05

**Authors:** Susana Pallarés, Eva Trinidad Gómez, África Martínez-Poveda, Manuel Miguel Jordán

**Affiliations:** 1 Carlos III Health Institute, 28029 Madrid, Spain; spallare@gmail.com; 2 Department of Agricultural and Environmental Sciences, Jaume I University, Campus Riu Sec s/n, 12071 Castellón, Spain; evatrinidadgomez@gmail.com; 3 Department of Agricultural Economics, Cartographic Engineering, Graphic Expression in Engineering, Miguel Hernández University of Elche, 03312 Orihuela (Alicante), Spain; africa.martinez@umh.es; 4 Department of Agrochemistry and Environment, Miguel Hernández University of Elche, 03202 Elche (Alicante), Spain

**Keywords:** indoor airborne particles, primary schools, statistical analysis, ceramic hub, Mediterranean basin

## Abstract

This study addresses the concentration of particulate matter and their size using a statistical analysis of data obtained inside seven schools located in the towns of Castellón (S1, S2, and S3), Alcora (S4, S5, and S6) and Lucena (S7) in northeast Spain. Samples were taken for five to eight hours, depending on school hours, to obtain a monthly sample for each school. The main goal of this study is to assess the differences depending on the type of location and the sampling point to be able to design corrective measures that improve the habitability and safety of the teaching spaces analyzed. The lowest concentrations of fine particulate matter, less than 2.5 µm, were registered at the rural location. The values of these particles found in industrial and urban locations were not substantially different. In the case of particulate matter between 2.5 and 10 µm, significant differences were observed between the three types of locations. The lowest concentrations of particles larger than 10 µm were registered at the rural location, and the highest concentrations were found at the industrial locations. Among the urban stations, the particle concentration of this fraction in station S2 was significantly higher than that in stations S1 and S3, which had similar concentrations. These values are also similar to those registered at school S6, which is at an industrial location. The resuspension of particles from both indoor sources as well as those transported from the outside is an important factor in the concentrations of particles inside classrooms.

## 1. Introduction

The concentration of particulate matter in the air inside a building is typically influenced by several factors such as the indoor generation of particles, the concentration of particles outside, the air exchange, the air exchange rate and the sedimentation characteristics of the particles [1,2]. Inside a building, there are a variety of sources of particles from heating, furniture, cleaning, resuspension, etc. [3]. However, in the absence of relevant indoor sources of emission, collected particles are strongly related to the exterior, and in some cases, the concentration of some indoors contaminants are similar or even greater than outdoors [4].

Similarly, to what happens in the outdoor atmospheric ambience, inside the buildings, particle size is determined by the sources of emission. The main origin of particle fractions smaller than 1 µm is the outdoor air, whereas the largest ones mainly come from activities conducted inside [5]. Coarse particles (larger than 2.5 µm) are generated inside buildings by activities such as cleaning, which can increase the resuspension of particles deposited on horizontal surfaces such as floors, carpets, and furniture [6,7]. The air exchange rate affects the time that particles remain indoors. Low exchange rates cause them to remain in the air for longer periods of time, which enables the accumulation of particles, therefore also leading to the registration of higher concentrations of indoor particles [8,9].

The effects of particulate contaminants on human health are determined by three factors: particle concentration, their size, and their chemical composition [3,5]. This study will address the first two aspects with the statistical study of particle concentration and size data, which have been obtained inside seven schools.

The selected schools have been located in the ceramic cluster of the province of Castellón (Northeast Spain). This industrial hub corresponds to a strategic area within the framework of air pollution control of the European Union (EU). Two ceramic clusters located in Modena (Italy) and Castellón (Spain) concentrate around 80% of the ceramic tile and glass-ceramic factories in the EU. Ceramic manufacturing can produce diffuse emissions of fluorine, carbon dioxide, sulfur and chlorine from the ceramic industry in these European industrial clusters with a significant risk to the environment and health [3]. In addition, in these places, there are significant contributions of natural origin, such as the climatic characteristics of the Mediterranean littoral basin. For example, winds have a great influence on the movement of pollutants in the atmosphere. On the coasts, the most important air currents are the periodic terrestrial air currents and sea breezes [5].

As children spend approximately one third of their time in schools, educatory environments deserve particular attention; however, the majority of research has focused on fine particles assessment in classrooms [9]. This manuscript aims to expand the study of particle concentrations from different sizes in primary schools by considering different indoor and outdoor school microenvironments [9]. The main goal of this study is to assess the differences depending on the type of location (urban, industrial, and rural) and the sampling point (conditioned by factors such as orientation, the morphology of the streets, ventilation, etc.) to be able to design corrective measures that improve the habitability and safety of the spaces analyzed.

In both cases, a statistical treatment composed of two phases was used to identify these differences. First, a descriptive study was conducted of the samples. Afterwards, an inferential analysis was conducted to detect the possible differences between the groups of data analyzed.

## 2. Materials and Methods

**Figure 1:**
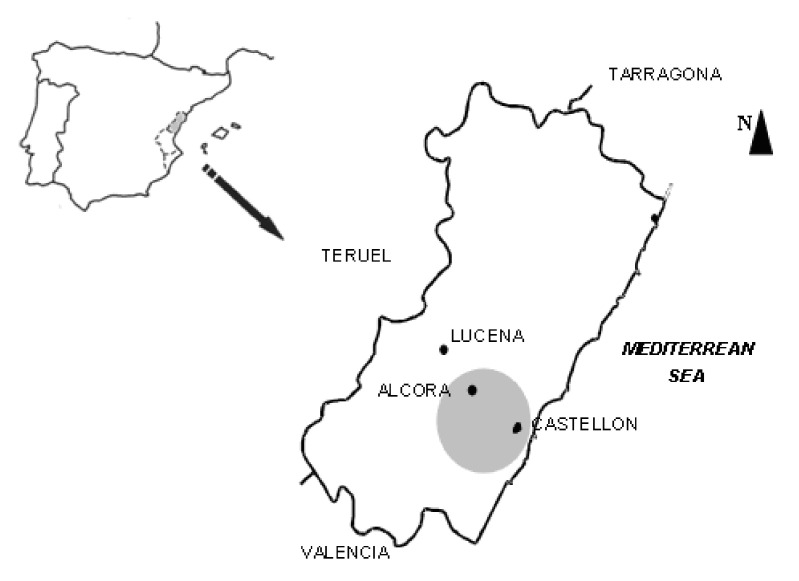
Location of the area studied (Scale 1:2,000,000).

This study was carried out in the province of Castllón, which starts from its capital, located in the Castellón plane—where there are no natural mountainous areas—passes through the town of Alcora, which sits on the fence of a mountain, and ends at Lucena, which is further from the coastal strip surrounded by mountains (Figure 1). These municipalities (Castellón, Alcora, and Lucena) represent three well differentiated environments: urban, industrial, and rural, respectively.

“Convenience sampling” was adapted for sample collection in this study [9]. Convenience sampling is not a probabilistic or random sampling technique used to obtain samples in seven schools located in the Spanish ceramic cluster according to ease of access, availability of teachers, children’s guardians, and center principals, in a given specific time interval with the relevant authorization of the local government. A total of 420 samples were obtained from three areas that in principle were very different regarding the quality of their air: the town of Castellón, which corresponds to an urban area, the town of Alcora, with the attributes of a ceramic industrial nucleus, and the town of Lucena, in a rural area.

Three schools (S) were selected in the town of Castellón: S1, S2, and S3. All of them were located near road networks with high traffic density. The three sampling stations are located in different neighborhoods of the town, in order to analyze whether the orientation with respect to the air currents and the different urban microenvironments influence the concentrations of indoor particles.

The three schools in the town of Alcora are close to an important ceramic belt. Three primary schools were chosen: S4, S5, and S6, at different points and with different orientations and settings.

In the municipality of Lucena, a rural town with low traffic density and low industrial concentration, the selected school was S7.

Table 1 summarizes the main characteristics of the seven schools studied. The ventilation system in all classrooms was manual.

The sampling period lasted twenty months (July and August excluded due to the holiday period in the schools). Samples were taken for five to eight hours, depending on school hours, to obtain a monthly sample for each school. In the sampling period, a total of 420 samples were collected (140 samples for each size fraction). However, it was necessary to select a much smaller representative number of samples to address the statistical analysis, avoiding systemic errors or affecting the results by uncontrollable variables (holidays, non-school days, power cuts, breakdowns, works or maintenance in classrooms, etc.). Sampling was performed using the RespiConTM device (Helmut Hund GmbH, Wetzlar, Germany) to collect the samples in indoor air using polytetrafluoroethylene (PTFE) filters (37 mm diameter). This device is a multi-stage virtual impactor that simultaneously collects ISO/CEN/ACGIH size fractions (inhalable, thoracic, and respirable particulate matter). RespiConTM traps particles in three individual collection filters using a flow rate of 3.11 L/min. A gravimetric study was performed to determine the collected mass using an analytical balance with a precision of 1 µg. The filters were kept at a temperature of 20 ± 1 °C and with (50 ± 5)% humidity controlled for 48 h, before being weighed. The particulate matter concentrations are shown in µg/m^3^ of aspirated air. Statistical analyses were conducted by the software SPSS Statistics v17.0 (SPSS Inc.: Chicago, IL, USA). The differences in particles concentrations were tested for significance among sampling locations and schools.

## 3. Results

### 3.1. Analysis of Differences Depending on the Type of Location

The first elements analyzed were the differences in the concentration levels of indoor particulate matter between the different types of locations. On one hand, an analytical and graphical descriptive study was conducted of each of the measured granulometric fractions (<2.5 µm, 2.5–10 µm, and >10 µm) at each of the three types of locations (urban, industrial, and rural) in order to analyze the possible differences between them.

On the other hand, an inferential analysis was conducted for the purpose of finding out whether the differences observed between the types of location, if any, were significant. These analyses enabled to generate models, inferences, and predictions related to the occurrences at hand considering the randomness of the observations [6].

#### 3.1.1. Fraction Smaller Than 2.5 µm

**Figure 2:**
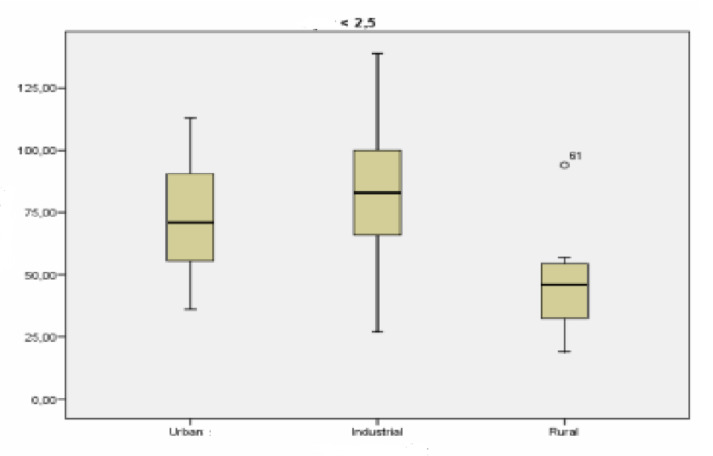
Box-plot of the concentration levels (in µg/m^3^) of particles smaller than 2.5 µm.

The Kolmogorov–Smirnov test was performed to study the normalcy of the variable analyzed in each surrounding. The results obtained in this test (asymptotic significance greater than 0.05) show a normal distribution at the three types of locations (urban, industrial, and rural). Figure 2 shows the concentration values of particles smaller than 2.5 µm at each type of location.

Differences can be seen between the locations on Figure 2, especially between the industrial and urban stations on one hand, and the rural one on the other. The industrial and urban locations have similar concentrations.

An inferential analysis was conducted of the concentrations particles smaller than 2.5 µm (PM2.5) for the purpose of finding out whether the differences observed in Figure 2 between the three types of locations were significant. Levene’s test was applied in first place in order to find out whether the location variances are homogeneous. The result obtained in this test (significantly greater than 0.05) makes it possible to verify the homogeneity of the variances.

The level of significance (sig.) obtained in the ANOVA procedure is lower than the figure selected as acceptable (0.05), which proves the existence of significant differences between the different types of locations (Table 2). A Bonferroni multiple comparison test (Table 3), which is applied in cases where there is homoscedasticity (homogeneity of variance), was performed in order to find out between which locations these differences existed.

The result of analyzing the variance supports the existing differences between stations located in industrial and rural areas. Higher values were always registered in industrial locations, whereas lower ones were present in rural locations.

#### 3.1.2. Fraction between 2.5 and 10 µm

First, the Kolmogorov–Smirnov test was applied to the concentrations of particles between 2.5 and 10 µm for each type of location (urban, industrial, and rural).

As with particles smaller than 2.5 µm, the result obtained with the Kolmogorov–Smirnov test (asymptotic significance greater than 0.05) showed that the analyzed variable (concentration of particles between 2.5 and 10 µm) followed a normal distribution in all types of locations.

**Figure 3:**
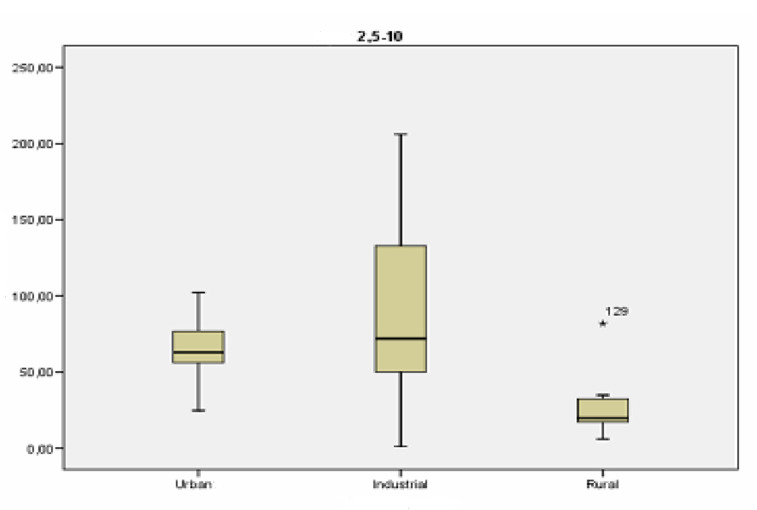
Box-plot of the levels of concentration (in µg/m^3^) of particles between 2.5 and 10 µm.

Figure 3 shows the concentration values of particles between 2.5 and 10 µm at each type of location and reveals clear differences between the types of locations, especially between urban and industrial stations and rural stations, which had lower concentration values of this granulometric fraction. A greater spread of data is registered for the industrial location, which had the highest mean values of concentration, possibly due the existence of significant differences among the schools located in this type of environment.

An inferential analysis of the concentration of particles between 2.5 and 10 µm variable was conducted in order to find out if the differences between the types of locations were significant. Levene’s test was applied in first place to find out if there was homogeneity of variance. The level of significance obtained with Levene’s test was less than 0.05, meaning that the variances were not homogenous. Thus, the variable was analyzed using a non-parametric approach, the Kruskal–Wallis test.

Table 4 shows the results obtained after applying the Kruskal–Wallis procedure. The asymptotic significance value rejects the hypothesis of the variable (concentration of particles between 2.5 and 10 µm) being equal at all three types of locations. Then, a multiple comparison analysis was performed in order to find out between which locations these differences existed. The study was conducted using Dunnett’s T3 test, as the condition of variance homogeneity was not met.

Table 5 shows, with an asterisk, between which locations there are significant differences—where the significance level is lower than 0.05. Differences are observed between the rural location, which represented a separate group with lower concentration values of particles between 2.5 and 10 µm, and the urban and industrial environments, which have similar concentration values.

#### 3.1.3. Fraction Greater Than 10 µm

First, a study of the normalcy of the distribution of the variable analyzed (concentration of particles greater than 10 µm) was conducted by applying the Kolmogorov–Smirnov test.

Once again it was observed that in all types of locations, the variable (concentration of particles greater than 10 µm) followed a normal distribution.

**Figure 4:**
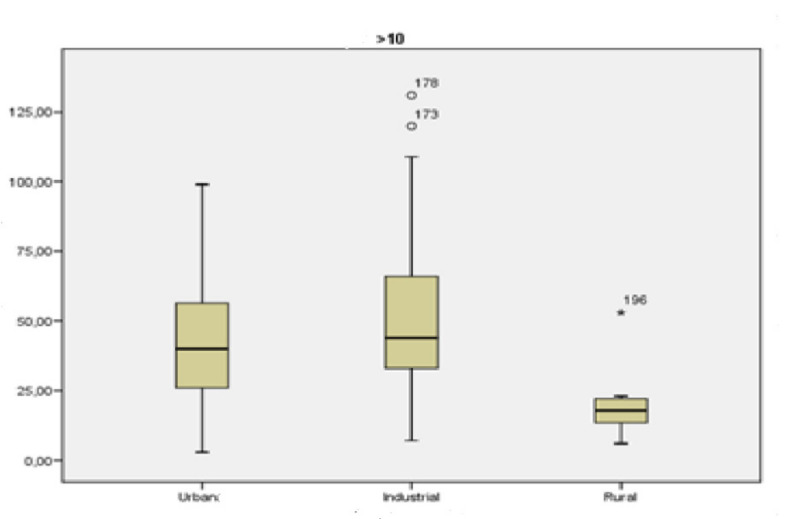
Box-plot of the concentration levels (in µg/m^3^) of particles greater than 10 μm.

Figure 4 shows the concentration values of particles greater than 10 µm at each type of location.

Figure 4 reveals differences between the types of locations, especially rural and urban locations compared to industrial locations. An inferential analysis of the data was conducted in order to analyze the significance of said differences.

In this case, Levene’s test rejected the homogeneity of variance, which made it impossible to apply an analysis of variance. The comparison of the three types of locations was solved using the non-parametric Kruskal–Wallis test (Table 6). This test, as the asymptotic significance value (less than 0.05) shows, makes it possible to reject the equality of the concentrations of particles larger than 10 μm at the three locations.

A multiple comparison analysis was performed using Dunnett’s T3 test for the purpose of finding out between which locations the significant differences existed because, as has been verified by applying Levene’s test, the variances were different (Table 7).

As in the case of particles between 2.5 and 10 µm, you can clearly differentiate between two groups of locations. On one hand, the one with lower values is the rural location. On the other are the two remaining locations, urban and industrial, which show values that are similar between them but significantly higher than the rural location.

### 3.2. Study of the Differences Depending on the Sampling Point

Regarding the variance of the concentration levels depending on the different sampling points (S1, S2, S3, S4, S5, S6, and S7), a descriptive analysis and an inferential analysis were also conducted, differentiating between the three particle size fractions. As in the prior case, the normalcy of the variable was studied in each school to begin with, because, due to the small amount of data available, distribution normalcy cannot be assumed. An inferential study was conducted afterwards.

#### 3.2.1. Fraction Smaller Than 2.5 µm

The Kolmogorov–Smirnov test was applied on a sample in order to assess the normalcy of the concentration of particles smaller than 2.5 µm variable. In all cases, the variable that was to be analyzed (the concentration of particles smaller than 2.5 µm) followed a normal distribution in all sampling points, given that the asymptotic significance value was greater than 0.05 in all cases.

**Figure 5:**
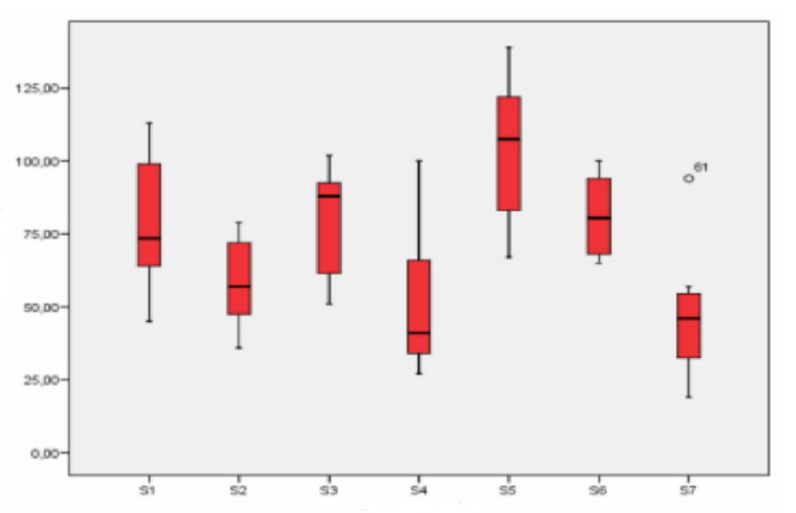
Box-plot of the concentration levels (in µg/m^3^) of particles smaller than 2.5 μm.

There were clear differences between the different sampling points (Figure 5). School S5 (industrial) showed the highest concentration values of particles smaller than 2.5 µm, and location S7 (rural) showed the lowest values. However, the latter were not too different from the values registered at industrial station S4.

Of the three schools in an industrial location, the highest concentration of particles smaller than 2.5 µm was registered at school S5, whereas the lowest values corresponded to S4.

Among urban schools, the one with the lowest concentration of this fraction was S2. Sampling stations S1 and S3 showed similar concentration values that were also similar to the ones registered at school S6, in the industrial location. The background values for rural station S7, while lower, were not much lower than those obtained at station S4. This fact shows that the rural station was noticeably influenced by the arrival of fine particles.

After verifying the normalcy of the distribution of the concentration data (Kolmogorov–Smirnov test), Levene’s test was applied to assess homogeneity of variance.

The results obtained after applying the ANOVA statistical procedure (significance lower than 0.05) showed the existence of significant differences among the seven sampling points (Table 8).

A multiple comparison study was conducted using the Bonferroni method, which was used when there is homogeneity of variance, in order to determine between which schools were the significant differences detected with the ANOVA procedure. The results of these multiple comparisons are shown on Table 9.

Table 9 flags with an asterisk the schools between which there are significant differences (significance lower than 0.05). Sampling point S7, which has the lowest values, is significantly different from S6 and S5, which are the locations with the highest concentrations of particulate smaller than 2.5 µm of all schools studied. Differences can also be established between sampling points S4 and points S5 and S6. Lastly, there are also differences between points S2 and S5.

#### 3.2.2. Fraction between 2.5 and 10 µm

The results obtained with the Kolmogorov–Smirnov test (asymptotic significance greater than 0.05) showed, as was the case with the concentrations of particles smaller than 2.5 µm, that the variable studied follows a normal distribution at all sampling points. If there is also homogeneity of variance (Levene’s test), it will be possible to apply an ANOVA procedure in the inferential analysis of the variable. Otherwise, a Kruskal–Wallis test will be applied.

**Figure 6:**
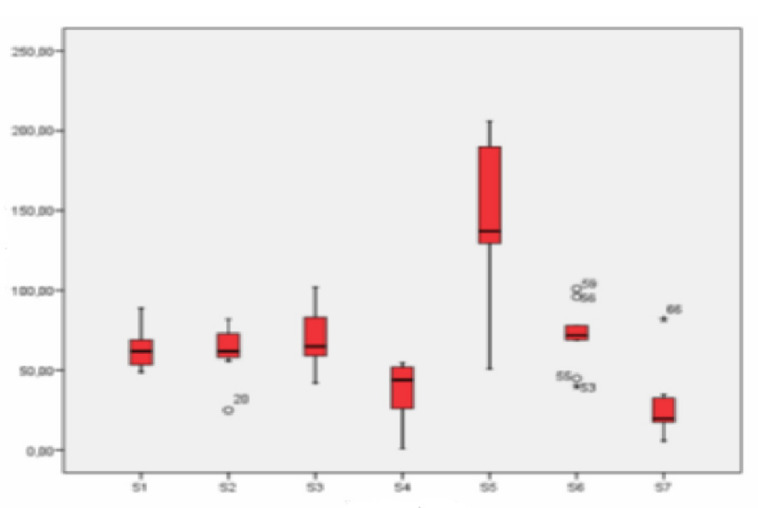
Box-plot of the concentration levels (in µg/m^3^) of particles between 2.5 and 10 µm.

The highest values clearly corresponded to school S5 (industrial location), and the lowest, to S7 (rural location). As observed in the graphical representation (Figure 6), station S5 had much higher concentration values of particles between 2.5 and 10 µm than all other points studied. The concentrations of the three schools in an urban environment (S1, S2, and S3) were similar, although station S3 had slightly higher values.

At the industrial location, the concentration values obtained for all three schools were very different. Whereas the levels of particulate between 2.5 and 10 µm obtained in station S5, as was mentioned, were the highest of all, school S6 showed similar concentrations to those obtained in the urban locations, and much lower values were registered in S4. Rural station S7 had the lowest concentration values. In this case, the difference with the urban and industrial stations was greater than for particulate smaller than 2.5 µm.

An inferential analysis of the variable was conducted after observing the differences in order to see whether these differences were significant. Levene’s test applied to the concentration data of particles between 2.5 and 10 µm showed that there was no homogeneity of variance. The non-parametric Kruskal–Wallis test was used to see whether there were significant differences between the schools studied. The results obtained are shown on Table 10.

The results obtained with the Kruskal–Wallis test (asymptotic significance lower than 0.05) showed that there were significant differences between the different sampling points. Dunnett’s T3 multiple comparison test (used when there is no homogeneity of variance) was conducted in order to determine between which schools these differences existed, thus comparing pairs of locations (Table 11).

Station S7, which showed the lowest values of particulate between 2.5 and 10 µm, can be considered similar to S2 and S4, and significantly different to the rest. Station S5, where the highest concentrations of this fraction were registered, can be considered significantly different to all other locations. School S6 can be considered different to S4 and S5 (both located in an industrial environment) and to S7 (rural), and similar to the three urban locations.

#### 3.2.3. Fraction Larger Than 10 µm

As in the case of all other studied particulate fractions, the Kolmogorov–Smirnov test was first applied to the data of the concentrations of particles larger than 10 µm for each of the seven schools. In all cases, as happened with the other two particulate fractions studied previously, the variable analyzed (concentration of particulate larger than 10 µm) follows a normal distribution.

**Figure 7:**
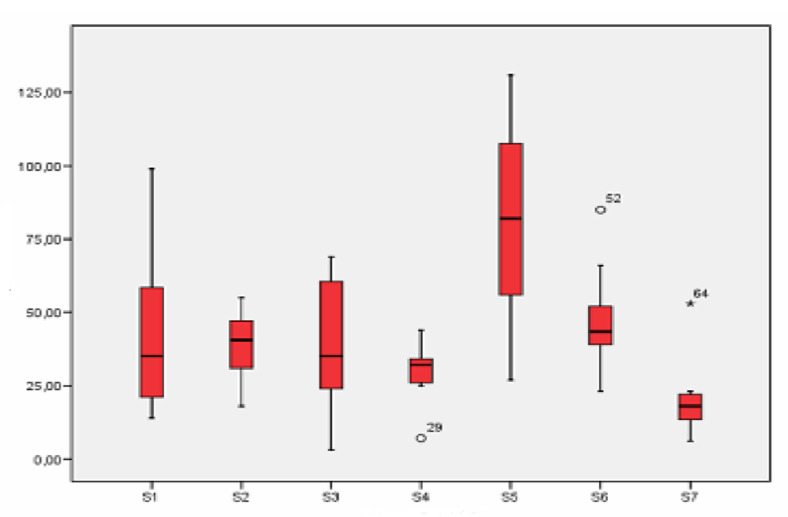
Box-plot of the concentration levels (in µg/m^3^) of particles greater than 10 μm.

Differences between the schools studied can be observed in Figure 7. Location S5 once again shows the highest values, whereas S7 has the lowest. This figure, shows that the three urban stations had similar values of this particulate fraction, although those in S1 were slightly higher.

Very different concentrations were registered in the three industrial stations, as with previous sections. School S5 had the highest values of the seven points studied, S6 has similar concentrations to urban stations and S4 has the lowest values. Rural school S7 yielded the lowest concentrations.

An inferential analysis of the concentration of particles larger than 10 μm was conducted in order to learn whether the graphical differences among the studied schools were significant.

The result obtained with Levene’s test discarded the hypothesis of variance homogeneity (significance is lower than 0.05), which means an ANOVA cannot be applied and a non-parametric test has to be used, in this case, the Kruskal–Wallis test.

The result obtained with this statistical test (Table 12) shows a lower level of significance than the one chosen as acceptable (0.05), which proves the existence of significant differences among the schools studied. After proving that there were significant differences between the sampling points, a multiple comparison analysis of pairs of schools was conducted to determine which these significant differences were between. Dunnett’s T3 test (Table 13) was used to do so, as it allows the comparison when variances are not equal.

The school with the lowest values is S7 and the one with the highest values is S5. In this case, S7 can be considered significantly different to S6 and S5. Differences were also found between S2 and S5 and S4 and S5. Thus, S5 was significantly different to the schools with the lowest values at each type of location: S2 in the urban environment, S4 industrial, and S7 rural.

### 3.3. Relationship between the Different Granulometric Fractions

This section looks at the relationship between the different granulometric fractions. To do so, the mean percentages of each particle size were calculated at each school studied.

In general, fine particles (smaller than 2.5 µm) were the most abundant in the various schools, representing 30–50% of the total concentration of particles gathered. It was observed that, of the urban stations, S1 and S3 had the highest proportion of particles smaller than 2.5 µm, which is believed to be due to these locations being heavily influenced by traffic, the main source of these types of particles in an urban environment [10].

The school located in a rural environment (S7) was the one with the finest particles, as practically half of the concentration of gathered particles were smaller than 2.5 µm. As mentioned, the differences between schools mainly lies in the influence that external sources exercise on indoor concentrations. In this case, although there was not a large number of sources of these particles outside, the concentration of particles smaller than 2.5 microns was the highest of all schools. The particulate is enriched by this fraction because there was a supply from areas further away from the sampling point. This fact is linked to small particles behaving similarly to a gas, remaining in the atmosphere for a long time, dispersing easier than coarse ones and even being able to travel through fluvial channels (Lucena river).

**Figure 8:**
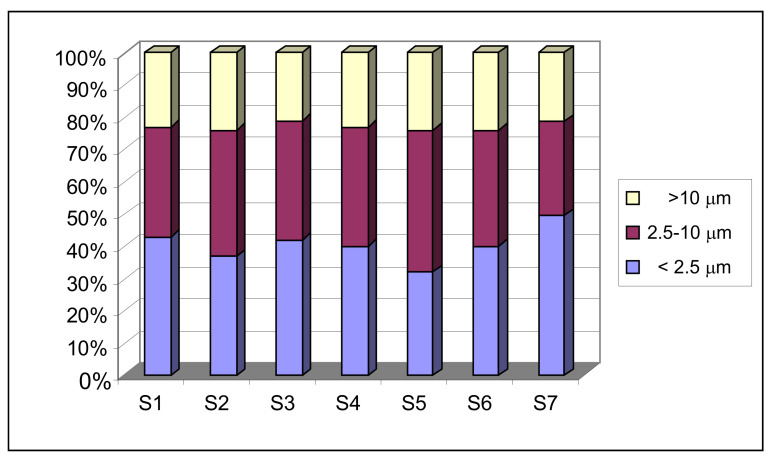
Percentages of the different granulometric fractions in the schools studied.

Figure 8 shows the percentages of the different granulometric fractions in the seven schools studied.

There were no considerable differences between urban and industrial stations except for school S5. The particulate gathered inside this sampling point was clearly enriched with the fraction between 2.5 and 10 µm. Section 3.2.3. of this study explained that the high values obtained in this particulate fraction were due to the noticeable contribution of material from the exterior through shoes and clothes due to the location of the school (a park that has not been asphalted next to the sampling point), the subsequent resuspension and accumulation of particles caused by activities conducted inside the classroom, and the greater influence of the direct supply of contaminants by the sea breeze when ventilating.

Table 14 shows that the Indoor-Outdoor ratio (I/O ratio) for PM10 was greater than unity in all sampling stations, indicating that there were statistically significant indoor sources of these particles, although this hypothesis must be validated. The I/O > 1 could also be explained by a transport event from the outside towards inside and not only for particle indoor sources.

## 4. Discussion

The indoor sources of fine particles (smaller than 2.5 µm) were generally associated to combustion processes such as heating [3]. As these sources do not change substantially from one type of location to another, the difference lies in the influence of outside air on the concentration values of particles of this fraction size. External sources were the main origin of fine particles, especially those smaller than 1 µm, inside buildings [5,11]. These particles penetrate more easily through narrow gaps than larger ones [12,13].

As expected, lower concentrations of fine particles were registered in the rural schools, as it is the type of location with the lowest number of external sources of emission.

The values of particles smaller than 2.5 µm found at industrial and urban locations were not substantially different, and no significant differences were found between them. The greatest standard deviation registered in the industrial environment reveals a greater dispersion of data, meaning it is possible that greater differences existed in the three schools of the industrial location. To verify this hypothesis and find out whether the type of environment is the only factor that impacts these differences, the same statistical treatment was applied to the seven schools separately without considering the type of location. The result of analyzing the variances backs the existing differences between stations located in industrial and rural areas. The highest values were always registered in the industrial area, and the lowest ones in the rural area.

In the case of particles between 2.5 and 10 µm, there were differences among the three types of locations. However, the statistical treatment reveals significant differences between schools located in the rural environment and those in industrial and urban environments. Once again, the rural location had the lowest concentration values of particles in this fraction, while the industrial one had the highest.

The resuspension of previously deposited particles due to the regular activities of a building’s inhabitants is an important factor in the indoor concentrations of particles. Thatcher and Layton [2] found that minor activities of four people or continuous walking by one inhabitant caused an increase of two to four times of the concentrations of particulate between 2.5 and 10 µm. This is why it was to be expected that in a school with a lower number of students (the one located in the rural station had between eight and 12 students, compared to the more than 20 who attend schools in all other locations), the resuspension was less impactful, and therefore the concentrations in the same conditions were lower in this sampling point. A high correlation was found in studies conducted on classrooms [11] between the number of students and the concentration of particles between 2.5 and 10 µm, which confirmed that human presence and the related activities represent an important source of coarse indoor particles. Luoma and Batterman [14] concluded in their study that activities in offices such as movement and working with paper increase the concentration of coarse particles.

As well as indoor particle emission sources (similar in all locations except for the rural location, where a lower emission is expected), there is another important source of coarse particles (greater than 2.5 µm): the dust brought in from outdoors through clothes and shoes [15]. Because of this, locations with a higher concentration of this particulate fraction outdoors or those where there is a higher number of particles of this size deposited in areas near the sampling point, are the ones where the highest indoor concentration values will be registered.

The lowest concentrations of particles larger than 10 µm were registered in the rural location, and the highest in the industrial location.

The main source of coarse indoor particles, as was mentioned in the section corresponding to the particulate fraction between 2.5 and 10 µm, was the dust carried by clothes and shows [11], which is deposited on the surfaces and is then resuspended. Therefore, the explanation of the differences observed between locations once again lies in the external concentrations of particulate larger than 10 µm, and the matter of this size located around the school centers of the different locations.

If we focus on the origin of the fine particles, they can either be from indoor sources (similar in all schools) or external sources. As mentioned previously, according to different authors [5,11], external sources are the main origin of particles smaller than 1 µm, meaning that the difference between schools resides in their amount and the influence that they have on these sources.

The three schools in the industrial location have noticeably different values. Sampling point S5 has the highest concentration values of particles smaller than 2.5 µm. The cause of these high levels could be the school’s own characteristics, such as the orientation and the morphology of its surrounding streets. This point is in an open location, with no topographical barriers or buildings that hinder the transportation of pollutants from the industrial area located to the east of this school by the marine component of the (diurnal) breeze. The S4 concentrations registered at are the lowest of the three industrial locations. The ventilation is the factor that has the greatest impact on these differences together with the structure of the building and orientation, as it is divided into classrooms with windows that lead out to an inner courtyard surrounded by adjacent buildings (an enclosed area with a shielding effect that stops the entrance of particles)—which decrease the concentrations of PM. This school, unlike the other studied points, is continuously exchanging air with the exterior, as one of the doors that leads to the terrace (of the room where the equipment is located) stayed open during sampling hours. Because of this constant flow, the air is renewed and there is no particle accumulation inside the school [8], as happens in the other studied schools, which results in S4 obtaining lower concentrations than expected. Poor ventilation is one of the factors that most contributes to indoor particulate.

Among the urban locations, the one with the lowest concentration of this fraction is point S2. This is linked to the location being less affected by traffic, one of the main sources of particles smaller than 2.5 µm in urban environments [10,16]. Non-mineral coal generated in combustion processes contributes by over 50% to the total concentration of this granulometric fraction in cities such as Madrid [17]. Sampling stations S1 and S3 show similar concentration values that are also similar to those registered in school S6, in an industrial location.

Despite being the lowest, the background values in the rural station were not much lower than those obtained in station S4. This proves that the rural station was noticeably influenced by the arrival of fine particles. This type of particles, especially those with an aerodynamic diameter smaller than 1 µm, have low sedimentation rates and, when dispersing, behave similarly to gases and steam [18,19,20]. Furthermore, the highest values of particles smaller than 2.5 µm in rural station S7 were registered in the month of June, thus being linked to the fact that, by behaving as a gaseous contaminant, its dispersion is heavily influenced by external factors such as the temperature. Its increase, together with solar radiation, facilitates the mobility of fine particles from the sources of emission through river valleys, in this case the riverbed of the Lucena river. Studies conducted in rural areas of the coastal Mediterranean area have also revealed an increase in the concentration of particles in warmer seasons [21].

The concentrations of particles between 2.5 and 10 µm from the three schools in an urban environment (S1, S2 and S3) are similar. However, the values of station S3 are slightly higher, which is believed to be due to the mineral particles from the resuspension of soil from a non-asphalted plot of land constantly used as a car park next to the school.

In the industrial locations, the concentration values obtained in the three schools are very different. Whereas the levels of particulate between 2.5 and 10 µm obtained in station S5 were the highest registered, as has been mentioned, school S6 showed concentrations similar to those obtained in urban locations, and much lower values were registered in S4.

When the windows are open, all sizes of particles penetrate the room with the same efficiency. However, when they are closed, as they have to enter through small grooves and holes, the finest particles make it inside more efficiently [22]. Ceramic companies generate particles, generally of mineral matter, with sizes between 2 and 100 µm, which would explain a greater concentration of these particles in the industrial location. However, the windows remain open in a majority of schools for short periods of time. The fact that there are increases in coarse particles is due to an added provision of particles of great importance: the transportation of matter from outside on shoes and clothes. Janssen [15] identified the resuspension from classroom activities as the most probable cause for high concentrations of PM10 and found that a majority of particles were dust brought in by shoes and clothes, and not due to combustion processes. In the case of S5, as well as being located in an area where the supply of matter is heightened due to its orientation favoring the dragging of contaminants by the sea breeze, it is located next to a park which all childhood education students who go to school have to cross, which significantly increases the supply of particles. This same reasoning makes it possible to explain the lower values obtained at station S4, which is located in a closed area on a first floor (lesser contribution by shoes) and which corresponds to a nursery school in which some infants barely walk. These characteristics decrease the provision of coarse particulate from the outside.

Meanwhile, the resuspension of particles from indoor sources as well as those carried in from outside due to the normal activities of a building’s inhabitants, is a key factor in the indoor concentrations of particles. Cleaning activities such as hoovering, dusting or sweeping also have an effect on particulate concentrations [23]. Different authors [11,24] observed in their studies that the number of inhabitants of a room and the duration of an activity increase the concentrations of coarse particles.

The effect that the provision of dust from outside through shoes and clothes has on the concentrations of particles between 2.5 and 10 µm, and the resuspension of particles generated by the activities of inhabitants of school S5 (which has the highest values), is reflected in the significant decrease of the concentrations of this granulometric fraction in the sample taken outside of class hours (uninhabited).

The lowest concentration of particles between 2.5 and 10 µm was registered in school S7 (rural location). This fact could mainly be due to two reasons. On one hand, the lesser concentration of this particulate fraction was found outside. On the other, the lesser number of students, who emit a smaller number of particles in their activities, which therefore causes the resuspension of particles to also be less significant. Furthermore, coarse particulate (larger than 2.5 µm) does not have the same ability to cross larger distances as fine particles, and thus the provision from more distant areas is lower.

The behavior of particles larger than 10 µm was very similar to that registered for particles between 2.5 and 10 µm. However, the values were lower than those registered in other fractions, as are the differences among stations.

Station S5 had the highest concentration values for particles larger than 10 µm, and station S7 once again has the lowest levels.

The main source of coarse indoor particles is material transported from outside through clothes and shoes, which is then deposited on various surfaces and will be resuspended. In a majority of cases studied, another source, to a lesser extent, are particles brought in by ventilation. Therefore, an explanation must be found for the differences observed among schools regarding the origins of outdoor particles larger than 10 µm. This type of particles mainly come from diffuse emissions. The transportation of goods produces diffuse emissions in processes such as dropping materials, the resuspension of particles on the road, and the wear of brakes and tires. The granulometry of these emissions is coarse, as over 60% are larger than 10 µm [25]. Extraction, movement, and storage activities also emit coarse particles [26].

School S5 once again showed the highest value, partly due to diffuse emissions having a greater impact on this sampling point, as it is located in an open area, which is conducive to the arrival of contaminants, and partly because of the existence of a park located next to the school, which also triggers the transportation of coarse particles (larger than 10 µm).

## 5. Conclusions

The lowest concentrations of fine particles (PM2.5) were registered at the rural station. The values of these particles found at industrial and urban locations were not substantially different. In the case of particles between 2.5 and 10 µm, there were differences between the three types of locations. However, there were significant differences between schools located in rural environments and those in industrial and urban environments. The lowest concentrations of particles greater than 10 µm were registered at the rural location, and the highest were registered at the industrial location. The explanation for the differences observed among locations resides in the outdoor concentrations of particulate larger than 10 µm. The stations located in the industrial area had different values, mainly due to the difference in the ventilation systems and their orientation.

Poor ventilation is one of the factors that most contributes to indoor particulate.

Thus, the resuspension of particles from both indoor sources as well as those transported from the outside is an important factor in the concentrations of particles inside classrooms.

The behavior of particles greater than 10 µm is very similar to the behavior of particles between 2.5 and 10 µm. However, the values are lower than those registered in other granulometric fractions

## Figures and Tables

**Table 1:** Main characteristics of the seven schools.

School	Site	Town Location	Traffic Density	Classroom Volume (m^3^)	Window Orientation
S1	Urban	E	High	268.03	WNW
S2	Urban	NW	Medium	159.56	SSE
S3	Urban	W	High	173.49	ESE
S4	Industrial	SE	Medium	36.17	WNW
S5	Industrial	E	Medium	109.09	ENE
S6	Industrial	SW	Low-Medium	97.67	SSE
S7	Rural	SE	Low	182.60	SE

**Legend:** S: school, E: East, NW: Northwest, W: West, SE: Southeast, SW: Southwest, WNW: West northwest, SSE: South southeast, ENE: East northeast.

**Table 2:** Results obtained with the ANOVA procedure.

Comparations	Sum of Squares	Df	Root Mean Square	F	Sig.
Inter-group	5946.22	2	2973.11	4.42	0.016
Intra-group	40,349.71	60	672.50		
Total	46,295.94	62			

**Legend:** Df: statistical differences; F: statistic inference F, Sig.: significance.

**Table 3:** Bonferroni multiple comparison test.

(I) Location	(J) Location	Mean Difference (I–J)	Standard Error	Sig.	95% Confidence Interval	
					Upper Limit	Lower Limit
Urban	Industrial Rural	−7.33 25.09	6.93 11.00	0.884 0.078	−24.41 −1.99	9.75 52.18
Industrial	Urban Rural	7.33 32.43 *	6.93 10.92	0.884 0.013	−9.75 5.53	24.41 59.33
Rural	Urban Industrial	−25.09 −32.43 *	11.00 10.92	0.078 0.013	−52.18 −59.33	1.99 −5.53

* The mean difference is significant at 0.05.

**Table 4:** Results obtained with the Kruskal–Wallis test.

Test Statistics a,b	
Chi-squared	12.458
Df	2
Asymp. sig.	0.002

a. Kruskal–Wallis test; b. Grouping variable: Location.

**Table 5:** Dunnett’s T3 multiple comparison test.

(I) Location	(J) Location	Mean Difference (I–J)	Standard Error	Sig.	95% Confidence Interval	
					Lower Limit	Upper Limit
Urban	Industrial Rural	−22.95 37.14 *	10.71 8.74	0.112 0.006	−49.81 11.82	3.90 62.47
Industrial	Urban Rural	22.95 60.10 *	10.71 13.20	0.112 0.000	−3.90 26.73	49.81 93.47
Rural	Urban Industrial	−37.14 * −60.10 *	8.74 13.20	0.006 0.000	−62.47 −93.47	−11.82 −26.73

* Mean difference is significant at 0.05.

**Table 6:** Results obtained with the Kruskal–Wallis test.

Test Statistics a,b	
Chi-squared	12.500
Df	2
Asymp. sig.	0.002

a. Kruskal–Wallis test; b. Grouping variable: Location.

**Table 7:** Dunnett’s T3 multiple comparison test.

(I) Location	(J) Location	Mean Difference (I–J)	Standard Error	Sig.	95% Confidence Interval	
					Lower Limit	Upper Limit
Urban	Industrial Rural	−13.94 19.51 *	6.98 6.42	0.144 0.021	−31.15 2.64	3.26 36.38
Industrial	Urban Rural	13.94 33.45 *	6.98 7.55	0.144 0.000	−3.26 14.26	31.15 52.64
Rural	Urban Industrial	−19.51 * −33.45 *	6.42 7.55	0.021 0.000	−36.38 −52.64	−2.64 −14.26

* Mean difference is significant at 0.05.

**Table 8:** Results obtained with the ANOVA procedure.

Comparations	Sum of Squares	Df	Mean Square	F	Sig.
Inter-groups	22,764.52	6	3794.09	9.03	0.000
Intra-groups	23,531.42	56	420.20		
Total	46,295.94	62			

**Table 9:** Bonferroni multiple comparisons.

(I) Location	(J) Location	Mean Difference (I–J)	Standard Error	Sig.	95% Confidence Interval	
					Lower Limit	Upper Limit
S1	S2 S3 S4 S5 S6 S7	20.37 0.42 28.54 −26.82 2.12 31.30	10.25 9.52 9.96 9.72 9.72 10.61	1.000 1.000 0.123 0.164 1.000 0.097	−12.25 −29.90 −3.16 −57.77 −33.07 −2.46	53.00 30.74 60.24 4.12 28.82 65.07
S2	S1 S3 S4 S5 S6 S7	−20.35 −19.95 8.17 −47.20 * −22.50 10.93	10.25 9.52 9.96 9.72 9.72 10.61	1.000 0.855 1.000 0.000 0.512 1.000	−53.00 −50.27 −23.54 −78.15 −53.45 −22.84	12.25 10.36 39.87 −16.25 8.44 44.70
S3	S1 S2 S4 S5 S6 S7	−0.42 19.95 28.12 −27.24 −2.54 30.88	9.52 9.52 9.21 8.96 8.96 9.91	1.000 0.855 0.073 0.075 1.000 0.061	−30.74 −10.36 −1.20 −55.75 −31.05 −0.66	29.90 50.27 57.45 1.26 25.96 62.43
S4	S1 S2 S3 S5 S6 S7	−28.54 −8.17 −28.12 −55.37 * −30.67 * 2.76	9.96 9.96 9.21 9.42 9.42 10.33	0.123 1.000 0.073 0.000 0.040 1.000	−60.24 −39.87 −57.45 −85.34 −60.64 −30.12	3.16 23.54 1.20 −25.39 −0.069 35.64
S5	S1 S2 S3 S4 S6 S7	26.82 47.20 * 27.24 55.37 * 24.70 58.13 *	9.72 9.72 8.96 9.42 9.17 10.10	0.164 0.000 0.075 0.000 0.195 0.000	−4.12 16.25 −1.26 25.39 −4.75 25.98	57.77 78.15 55.75 85.34 53.88 90.28
S6	S1 S2 S3 S4 S5 S7	2.12 22.50 2.54 30.67 * −24.70 33.43 *	9.72 9.72 8.96 9.42 9.17 10.10	1.000 0.512 1.000 0.040 0.195 0.034	−28.82 −8.44 −25.96 0.69 −53.88 1.28	33.07 53.45 31.05 60.64 4.48 65.58
S7	S1 S2 S3 S4 S5 S6	−31.30 −10.92 −30.88 −2.76 −58.13 * −33.43 *	10.61 10.61 9.91 10.33 10.10 10.10	0.097 1.000 0.061 1.000 0.000 0.034	−65.07 −44.70 −62.43 −35.64 −90.28 −65.58	2.46 22.84 0.66 30.12 −25.98 −1.28

* Mean difference is significative at 0.05.

**Table 10:** Kruskal–Wallis test for the concentrations of particulate between 2.5 and 10 μm.

Test Statistics a,b	
Chi-squared	38.743
Df	6
Asymp. sig.	0.000

a. Kruskal–Wallis test; b. Grouping variable: Location.

**Table 11:** Dunnett’s T3 multiple comparison test.

(I) Location	(J) Location	Mean Difference (I–J)	Standard Error	Sig.	95% Confidence Interval	
					Lower Limit	Upper Limit
S1	S2 S3 S4 S5 S6 S7	0.97 −6.45 27.08 −83.00 * −7.96 35.14 *	6.96 6.77 8.26 14.42 7.18 9.15	1.000 0.999 0.098 0.002 0.994 0.049	−23.87 −29.89 −3.19 −136.74 −33.29 −0.12	25.81 16.98 57.35 −29.26 17.36 70.16
S2	S1 S3 S4 S5 S6 S7	−0.97 −7.42 26.11 −83.97 * −8.93 34.17	6.96 7.94 9.24 15.01 8.29 10.04	1.000 0.999 0.191 0.002 0.996 0.078	−25.81 −35.02 −6.68 −138.52 −37.88 −2.54	23.87 20.17 58.91 −29.42 20.01 70.88
S3	S1 S2 S4 S5 S6 S7	6.45 7.42 33.54 * −76.55 * −1.51 41.59 *	6.77 7.94 9.10 14.92 8.13 9.91	0.999 0.999 0.037 0.004 1.000 0.019	−16.98 −20.17 1.41 −130.89 −29.54 5.38	29.89 35.03 65.66 −22.20 26.52 77.80
S4	S1 S2 S3 S5 S6 S7	−27.08 −26.11 −33.54 * −110.08 * −35.04 * 8.06	8.26 9.24 9.10 15.65 9.40 10.98	0.098 0.191 0.037 0.000 0.033 1.000	−57.35 −58.91 −65.66 −165.87 −68.15 −31.23	3.19 6.68 −1.41 −54.29 −1.94 47.34
S5	S1 S2 S3 S4 S6 S7	83.00 * 83.97 * 76.54 * 110.08 * 75.04 * 118.14 *	14.42 15.01 14.92 15.65 15.11 16.14	0.002 0.002 0.004 0.000 0.004 0.000	29.26 29.42 22.20 54.29 20.36 61.03	136.74 138.52 130.89 165.87 29.71 75.24
S6	S1 S2 S3 S4 S5 S7	7.96 8.93 1.51 35.04 * −75.04 * 43.10 *	7.18 8.29 8.13 9.40 15.11 10.19	0.994 0.996 1.000 0.033 0.004 0.017	−17.36 −20.01 −26.53 1.94 −129.71 6.17	33.27 37.88 29.54 68.15 −20.36 80.03
S7	S1 S2 S3 S4 S5 S6	−35.14 * −34.17 −41.59 * −8.06 −118.14 * −43.10 *	9.15 10.04 9.91 10.98 16.14 10.19	0.049 0.078 0.019 1.000 0.000 0.017	−70.16 −70.88 −77.80 −47.34 −175.24 −80.03	−0.12 2.54 −5.39 31.23 −61.03 −6.17

* Mean difference is significant at 0.05.

**Table 12:** Kruskal–Wallis procedure for the concentration of particulate larger than 10 µm.

Test Statistics a,b	
Chi-squared	25.614
Df	6
Asymp. sig.	0.000

a. Kruskal–Wallis test; b. Grouping variable: Location.

**Table 13:** Dunnett’s T3 multiple comparison test.

(I) Location	(J) Location	Mean Difference (I–J)	Standard Error	Sig.	95% Confidence Interval	
					Lower Limit	Upper Limit
S1	S2 S3 S4 S5 S6 S7	4.00 3.20 13.19 −37.61 −4.85 22.00	10.90 12.05 10.67 14.22 11.44 11.24	1.000 1.000 0.972 0.254 1.000 0.662	−38.66 −40.92 −29.33 −87.52 −48.00 −21.01	46.66 47.33 55.71 12.29 38.30 65.01
S2	S1 S3 S4 S5 S6 S7	−4.00 −0.80 9.19 −41.61 * −8.85 18.00	10.90 7.83 5.46 10.88 6.84 6.49	1.000 1.000 0.829 0.036 0.973 0.219	−46.66 −28.38 −10.39 −81.16 −33.00 −5.48	38.66 26.79 28.78 −2.07 15.30 41.48
S3	S1 S2 S4 S5 S6 S7	−3.20 0.80 9.99 −40.82 −8.05 18.80	12.05 7.83 7.50 12.03 8.55 8.28	1.000 1.000 0.965 0.060 0.999 0.452	−47.33 −26.79 −16.69 −82.72 −37.56 −10.16	40.92 28.38 36.67 1.09 21.45 47.75
S4	S1 S2 S3 S5 S6 S7	−13.19 −9.19 −9.99 −50.81 * −18.04 * 8.80	10.67 5.46 7.50 10.64 6.46 6.09	0.972 0.829 0.965 0.008 0.202 0.931	−55.71 −28.78 −36.67 −89.96 −40.98 −13.42	29.33 10.39 16.69 −11.65 4.89 31.03
S5	S1 S2 S3 S4 S6 S7	37.61 41.61 * 40.82 50.81 * 32.76 59.61 *	14.23 10.88 12.03 10.64 11.41 11.21	0.254 0.036 0.060 0.008 0.177 0.002	−12.29 2.07 −1.08 11.65 −7.74 19.41	87.52 81.16 82.72 89.96 73.27 99.81
S6	S1 S2 S3 S4 S5 S7	4.85 8.85 8.05 18.04 −32.76 26.85 *	11.44 6.84 8.55 6.46 11.41 7.35	1.000 0.973 0.999 0.202 0.177 0.039	−38.30 −15.30 −21.45 −4.89 −73.27 0.94	48.00 33.00 37.56 40.98 7.74 52.76
S7	S1 S2 S3 S4 S5 S6	−22.00 −18.00 −18.80 −8.80 −59.61 * −26.85 *	11.24 6.49 8.28 6.09 11.21 7.35	0.662 0.219 0.452 0.931 0.002 0.039	−65.01 −41.48 −47.75 −31.03 −99.81 −52.76	21.01 5.48 10.16 13.42 −19.42 −0.94

* Mean difference is significant at 0.05.

**Table 14:** Indoor and outdoor PM10 concentrations obtained and I/O ratios.

School	Indoor PM10 Conc. Calcul.—μg m^−3^—(min–max)	Outdoor PM10 Conc.—μg m^−3^—(min–max)	I/O Ratio
S1	132 (95–160)	53 (30–73)	2.6
S2	114 (63–138)	54 (36–81)	2.3
S3	141 (92–185)	59 (29–98)	2.6
S4	69 (19–92)	53 (35–76)	1.3
S5	261 (201–309)	36 (17–49)	7.8
S6	145 (98–181)	45 (33–68)	3.5
S7	71 (15–159)	36 (16–52)	2.1

**Legend:** Conc. Calcul: concentrations calculated, min-max: minimum-maximum I/O: Indoor/Outdoor.

## Data Availability

Data available on request from the authors.
